# Corneal Endothelial Characteristics in Normal Chinese Han Children and Youngsters: A Study from the Specular Microscopy Descriptions

**DOI:** 10.1155/2022/5338725

**Published:** 2022-05-20

**Authors:** Lilian Xie, Huilong Fang, Yuyu Xie, Haiyan Wang, Ru Liu, Zhiyuan Li, Jundong Zhu

**Affiliations:** ^1^The Ophthalmology Department of the Affiliated Chenzhou Hospital, Hengyan Medical School, University of South China, Chenzhou, Hunan, China 423000; ^2^Department of Pathogenic Biology and Immunology, Xiangnan University, Chenzhou, Hunan, China 423000; ^3^Graduate School of Medical School of Shanghai Jiaotong University, Shanghai, China 200025; ^4^The Operation Department of the Affiliated Chenzhou Hospital, Hengyan Medical School, University of South China, Chenzhou, Hunan, China 423000; ^5^The Diagnosis and Treatment Technology Research and Development Centre for Dry Eye and Ocular Surface Disease of Chenzhou, Chenzhou, Hunan, China 423000; ^6^Changsha Aier Eye Hospital, Changsha Hunan, China 410000

## Abstract

**Objective:**

To observe the morphological changes of corneal endothelial cells in healthy Chinese children and youngsters and analyze the sensitive and specificity of the endothelial assessments.

**Methods:**

14,670 Chinese healthy volunteers enrolled were examined by specular microscopy, and the endothelial descriptive indexes: the central corneal thickness (CCT), endothelial cell density (ECD), coefficient of variation in average cell size (CV), the percentage of regular hexagonal cells (hexagonality, HEX), cell size of minimal cell (*S*_min_), cell size of maximal cell (*S*_max_), average cell size (*S*_avg_), and size of standard deviation of cell area (*S*_sd_) as well as sex and age were analyzed.

**Results:**

The average age of this study is 17.36 ± 7.58 (4–30) years. There is no sex predominance: 7,260 male (49.5%) and 7,410 female (50.5%). The mean CCT, ECD, CV, HEX, *S*_min/max_, *S*_avg_, and *S*_sd_ are 529.94 ± 31.53 (437–644) *μ*m, 3,051.28 ± 375.49 (2,031–4,074) cells/mm^2^, 28.34 ± 4.36 (18–40) %, 61.21 ± 10.29 (17–89) %, (147.79 ± 21.94 to 678.29 ± 120.96) *μ*m^2^, 332.74 ± 44.62 *μ*m^2^, and 95.02 ± 23.17 *μ*m^2^, respectively. The CCTs keep consistency. The ECD decreased rate is 1.02%/year. The curve of ECD and hexagonality expresses the same linear tender. The CCT and endothelial evaluation indexes have no sex predominant (*p* > 0.05); the quantitative indicators: CCT, ECD, and HEX are significant negative associated with age (*p* = 0.001 or *p* < 0.001); the variability indexes: the CV, *S*_min_, *S*_max_, *S*_avg_, and *S*_sd_ are positive correlation (*p* < 0.001). The coefficients of CCT, HE, and *S*_min_ are -0.35, -0.59, and 1.17, respectively.

**Conclusions:**

The ECD decrease rate is 1.02%/year of the normal Chinese Han childhood to the earlier adulthood. The ages 4 to 12, 13 to 20, and 21 to 30 can be named as the childhood, puberty and adulthood from endothelial biologic identity. The HEX is the sensitivity marks for the polymorphisms while the S _min_ is the specificity indicator CVs upon the Topcon Noncon Specular microscopy results.

## 1. Introduction

Human corneal endothelial cells (hCECs), originated from the neural crest, fulfill the posterior surface of the cornea and are construct of a monolayer of overlapping cells arranged in a mosaic pattern of mostly hexagonal appearance [1]. These metabolically lively cells are responsible for adjusting fluid and solute conveying between the aqueous humor and corneal stoma so as to preserve normal corneal thickness and transparency [2]. Not as the epithelia, the hCECs cannot regenerate and the density declining throughout the entire human life [2, 3]. Other factors that contribute to alteration of CEC morphology and quantity include injury [2], contact lens wearing [4], dry eye [5], intraocular surgery [6], and systemic diseases such as diabetes mellitus [7].

As a result, in cases when the age grows, the endothelial cell density (ECD) and the percentage of hexagonality (HEX) of these cells have decreased, with a corresponding proportional increase in the average cell area (polymorphism) [8]. Repair of hurts to corneal endothelial cells occurs by extension, migration, and amitotic nucleus cleavage of the residual endothelial cells. Therefore, parameters such as the amount, pleomorphism, and hexagonality of these cells are used to evaluate the capacity of endothelial cells [9].

Overall analysis of the corneal endothelial can be executed with a specular microscopy that distracts a slim light onto the corneal tissue surface and gathers the reflected light through a film plane. The central corneal thickness (CCT, *μ*m), mean endothelial cell density (ECD, cell/mm^2^), coefficient of variation of cell area (CV), percentage of hexagonality cell (HEX), and mean cell area (AVG, *μ*m^2^) are measured by the specular microscopy [10] so as to delusive these distinctive features.

Anyway, there is still lack of evidence to clarify the human endothelial cell transmutation of normal Chinese Han childhood, puberty, or adulthood. There are routine eight evaluation indicators by Noncon Specular microscopy (SP-1P, Topcon Corporation), and the sensitive and specificity of each symbols should be clarified.

## 2. Materials and Methods

### 2.1. General Data and Schedule

After obtaining informed consent, 14,670 volunteers (14,670 eyes, right eye) were performed with Snellen VA, autorefraction, slit-lamp biomicroscopy, conjunctival and corneal fluorescein staining, fundus examination, and IOP assessment. Amount and sharp of endothelial were examined using a noncontact specular microscopy (Topcon Noncon Specular microscopy, SP-1P; Topcon Corporation, Tokyo, Japan) at the Changsha Aier Eye Hospital (Group), Central South University and the First People's Hospital of Chenzhou, Hengyang Medicine School, South China University from January 2021 to December 2021. Each age group included the following: 4-8, 9-12, 13-16, 17-20, 21-25, and 26-30 years old. Endothelial cell measurements were performed with the autofocusing model at the central cornea point with 4 mm diameter area and autocell selection, and analysis was applied in the examination.

**Figure 1:**
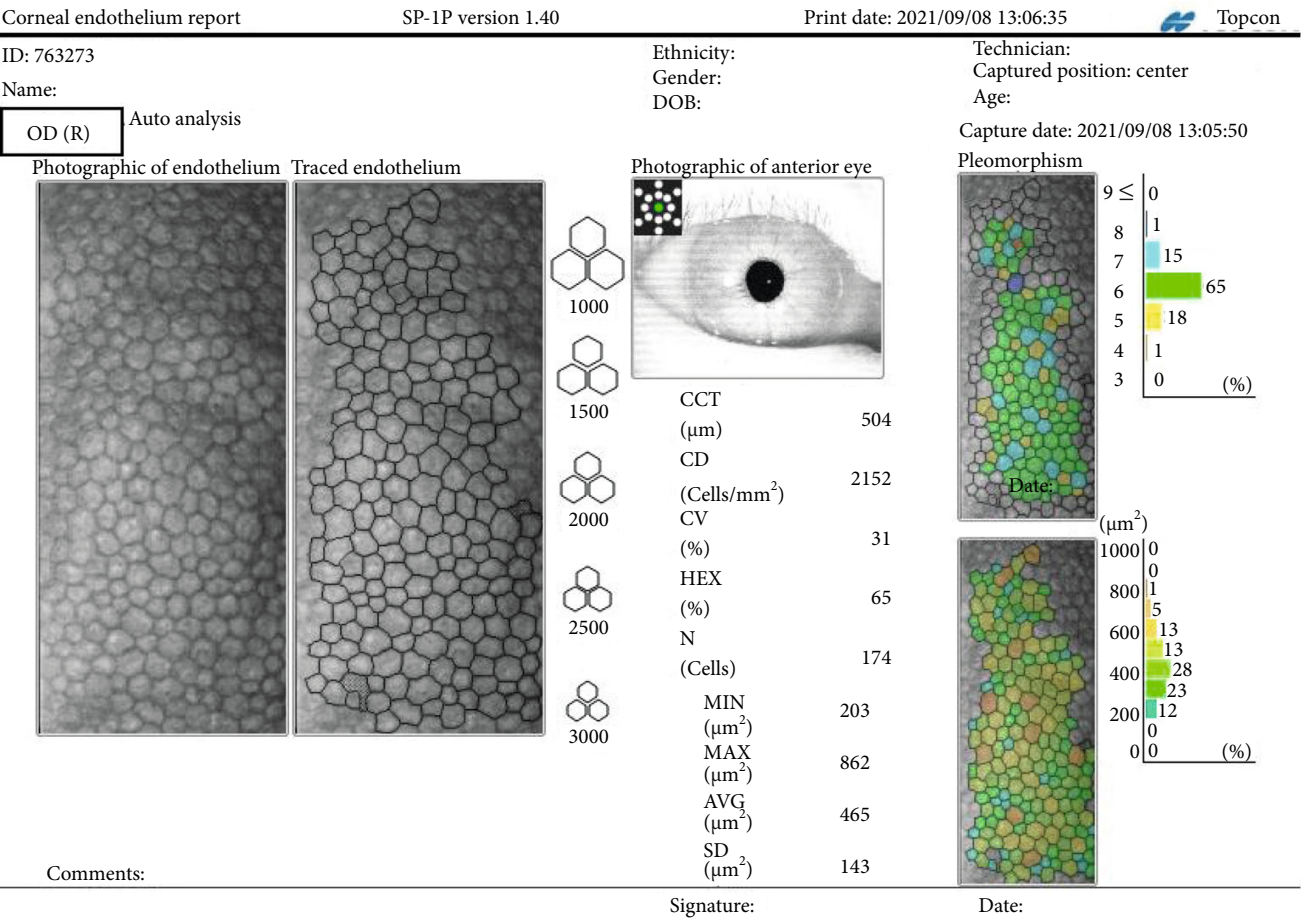
The schematic diagram for human corneal endothelial capture by SP-1P.

The main corneal parameters were as follows: the central corneal thickness (CCT), endothelial cell density (ECD), coefficient of variation in average cell size (CV), the percentage of regular hexagonal cells (hexagonality, HEX), cell size of minimal cell (*S*_min_), cell size of maximal cell (*S*_max_), average cell size (*S*_avg_), and size of standard deviation of cell area (*S*_sd_).These eight parameters of endothelial layer were analyzed by the SP-1P machine built-in software (Figure 1).

### 2.2. Inclusion and Exclusion Criteria

Patients' age and sex were also noted. All the patients were Chinese Han nationality. The patients were examined by routine slit lamp and ophthalmoscope.

The exclusion criteria were as follows: diabetes mellitus, corneal, and intraocular diseases including keratitis, glaucoma, or using antiglaucoma medications, corneal disorders (i.e., ectasia, scar, dystrophy, and dry eye with ocular surface fluorescein staining), congenital cornea diseases, congenital iris disorder and congenital cataract, pterygium that margin into cornea > 2 mm, recent ocular infection, previous ocular surgery or ocular trauma, history of contact lens wear within 14 days, and refractive errors with spherical equivalent beyond ±6 diopters.

### 2.3. Project Design

The age of all subjects ranged from 4 to 30 years, and subjects were allocated into six groups stratified by age. The volunteers' distribution is as follows: 4–8 years (2,080 eyes, 2,080 patients), 9–12 years (3,010 eyes, 3,010 patients), 13–16 years (2,040 eyes, 2,040 patients), 17–20 years (1,930 eyes, 1,930 patients), 21–25 years (2,720 eyes, 2,720 patients), and 26–30 years (2,890 eyes, 2,890 patients). The number of men and women in each group was nearly matched. Only the data of the right eye was used for demonstrating the relationship between age, corneal CCT, and endothelial parameters. This model was chosen because the ECD of each volunteer's eye was repeatedly measured for the eligible data.

The main corneal parameters were calculated, and results were compared among these groups. Correlations between the main corneal parameters (CCT, ECD, CV, HEX, *S*_min_, *S*_max_, *S*_avg_, and *S*_sd_) and age were found.

### 2.4. Medical Ethics

This study followed the principium of the Declaration of Helsinki, and this protocol was approved by the institutional review board of the ethnic committee of the Changsha Aier Eye Hospital (group) (ID: 2021KYYJ002).

### 2.5. Statistical Analysis

The majority of data did not have a normal distribution; thus, nonparametric tests were adopted. Statistical analyses were performed using SPSS for Windows IBM SPSS® 23.0 (SPSS Inc., Chicago, IL, USA). Student's pair *t*-test and Pearson's correlation coefficient (*r*) values were calculated. ANOVA test was used to compare the eight parameters of endothelial cells among study groups. The categorical variables use Fisher exact or chi-square test. Multiply linear regressions modeling analysis was used to estimate the corneal ECD over time and between each stage. Both vicariate and multivariate analyses were performed to estimate unadjusted and adjusted effects. All predictors with *p* < 0.25 in the vicariate models were considered sufficiently important for consideration. The exception to this rule was ECD, the study effect, which was forced into the multivariable model regardless. A value of *p* < 0.05 was considered as statistically significant.

## 3. Results

The mean age of participants in this study was 17.36 ± 7.58 (range, 4–30) years. There was no sex predominance: 7,260 male (49.5%) and 7,410 female (50.5%).The measurements of all parameters in all studied eyes were shown in Table 1. The mean CCT was 529.94 ± 31.53 (range, 437–644) *μ*m. Mean ECD was 3,051.28 ± 375.49 (range, 2,031–4,074) cells/mm^2^. Mean CV was 28.34 ± 4.36 (range, 18–40) %. Mean HEX (%) was 61.21 ± 10.29 (range, 17–89) %. *S*_min_ was 147.79 ± 21.94 *μ*m^2^. *S*_max_ was 678.29 ± 120.96 *μ*m^2^. *S*_avg_ was 332.74 ± 44.62 *μ*m^2^. *S*_sd_ was 95.02 ± 23.17 *μ*m^2^.

**Figure 2:**
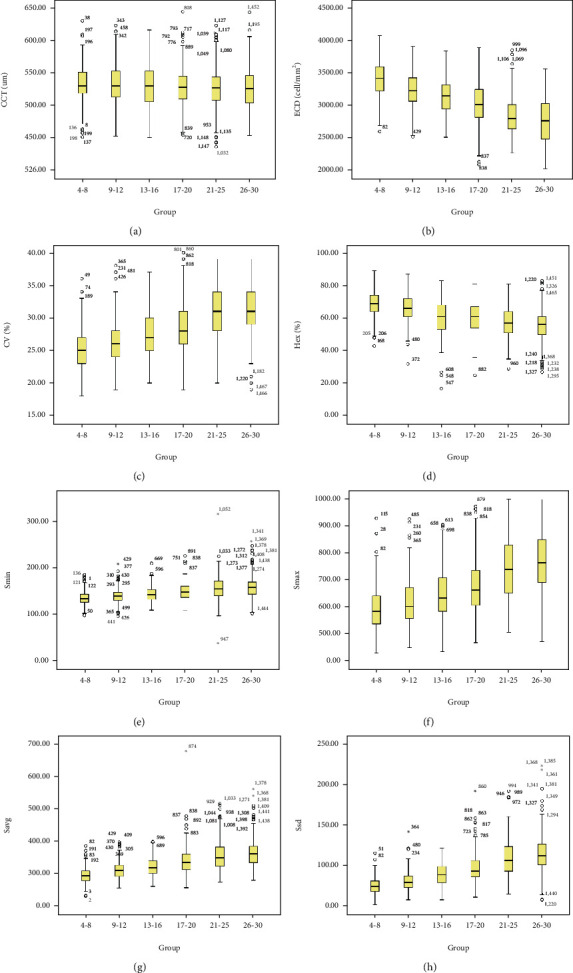
Eight parameters in different study groups. (a) Central corneal thickness. (b) Endothelial cell density. (c) Coefficient of variability. (d) Hexagonality. (e) Size of minimal cell. (g) Average cell size. (h) Size standard deviation.

Table 2 and Figure 2 the parameters of corneal endothelial in different age groups of normal Chinese children, 221 youngsters and adults were shown in Table 2 and Figure 2.

**Figure 3:**
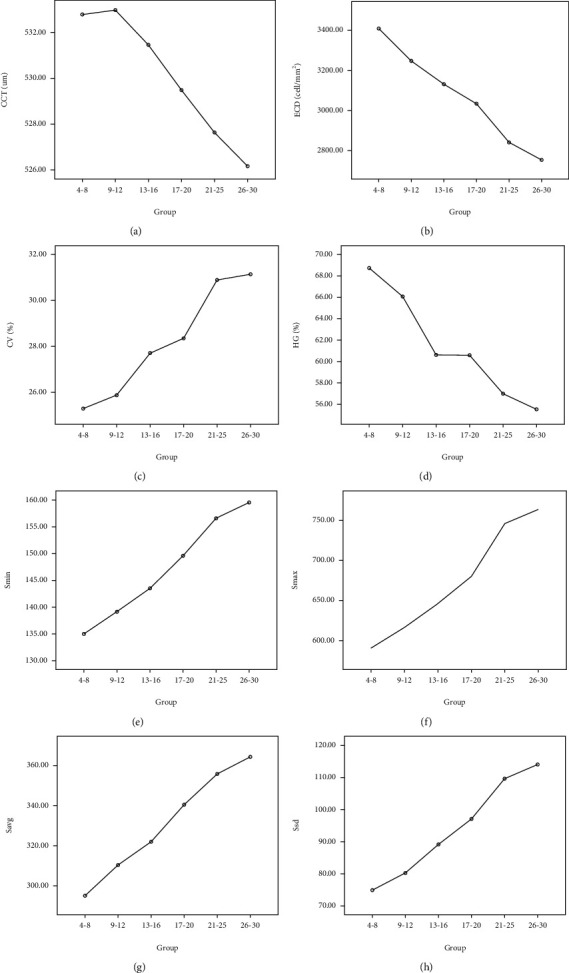
Eight parameters in different study groups. (a) Central corneal thickness. (b) Endothelial cell density. (c) Coefficient of variability. (d) Hexagonality. (e) Size of minimal cell. (g) Average cell size. (h) Size standard deviation.

The ranges of the eight parameters in 6 groups were demonstrated in Figure 3. CCT in different groups has no difference (*p* = 0.061), but the ECD quantity index combined with those endothelial morphology descriptive index such as HEX, CV, *S*_min_, *S*_max_, *S*_avg_, and *S*_sd_ for endothelial demonstrates these obvious difference according to age criterions (*p* < 0.001).

The CCT at the same level is upon the aged groups; the ECD has the decreased rate as 1.02%/year. Our data also show that the ECD decrease rate is different according to those childhood and youngster observers, and the childhood stage figure as 1.26%/year shows the fierce stage of endothelial declined so that this stage should keep stable without outside spur or noxious; meanwhile, the youngsters' yearly ECD decrease is 0.298% which is the most sluggish stage of our targeted volunteers. The curve of ECD and HEX manifested the same crests and troughs tender.

The CCT and endothelial characteristics from both qualitative and quantitative evaluation index between male and female in normal Chinese Han children and youngsters in different groups have no difference (*p* > 0.05) (Table 3).

All the data analyzed by ANOVA test indicated that eight parameters of corneal thickness and endothelial characteristics were significantly different among different age groups (Table 4). The quantitative indicators such as the CCT, ECD, and HEX had the significant negative association with age by multivariate analysis (*p* = 0.001 or *p* < 0.001). Meanwhile, the corneal endothelial variability index such as the CV, *S*_min_, *S*_max_, *S*_avg_, and *S*_sd_ showed positive correlation (*p* < 0.001).

### 3.1. The Multivariate Regression Equation Analysis between Age and Different Parameters Was Shown in Figure 4

The coefficient of variation (CV) of CCT is -0.35, slight negative association; the coefficient of variation (CV) of ECD is -30.31 which is the obvious negative correlation index; the coefficient of variation (CV) of CV is 0.29 which is a normal index; the coefficient of variation (CV) of HE is -0.59 which is a bit exaggerated indication upon this regress line equation.

## 4. Discussion

### 4.1. The Physiology of Corneal Endothelial Cells

On the corneal posterior surface, a single film of corneal endothelial cells (CECs) is constructed in a tightly packed tessellated pattern on its basement membrane, the Descemet membrane (DM), which forms a baffle between the corneal stroma and anterior aqueous chamber [11], depending on the species, the corneal epithelium, the corneal endothelium, or both have a major role in regulating corneal hydration [12]. CECs are metabolically active and accelerate a continuous drawing action with a fluid-coupled active efflux of ions from the corneal stroma into the aqueous humor by ionic channel active transport [13].

### 4.2. The Fundamental Process of Endothelial Variations

In vivo, postpartum human CECs are terminally differentiated (TD), nonmitotic organs, which are static in the G1 phase of the cell cycle [14]. A plurality of factors, including cell-cell contact inhibition, the exits of cell cycle negative regulators (e.g., cell cyclin-dependent kinase inhibitors, p15INK4b, and p27kip1), growth inhibitors (e.g., transforming growth factor-*β* and TGF-*β*) immersing in the aqueous humor, and stress-induced precocious senescence, contribute to the retention of CECs in their nonregenerative conformation [15, 16]. The mean CEC density is the highest of the neonates (at 3,000-4,000 cells/mm^2^), decreases gently at the promised rate of about 0.3-0.6% per annum, and comes to ~2,800 cells/mm^2^ in adolescence and~2,000 cells/mm^2^ in elder [17, 18]. Previous literatures have the conclusion that with the age growth, the human corneal endothelial becomes hexagonal morphologically irregular and may become easier permeability to fluorescein staining [19].

### 4.3. The Characteristic of 4-30 Years Chinese's Endothelial Count

According to our 4-30 years period of Chinese volunteers' cell biological measurements, these data also show that the ECD has the decreased rate as 1.02%/year which is higher than previous studies [20–22]. Ought to the targeted observes from the childhood to the earlier adults' popular selection or statistical sample size bias, the ECD decrease rate of childhood stage figure is 1.26%/year which states that this is the fierce stage of endothelial declined so that this 4-12 years childhood phase should be shielded from outsider spurs or noxious as least as possible; meanwhile, the youngsters' (13-20 years old) yearly ECD decrease is 0.298% which is the steadiest stage of our participants. The graph of both ECD and hexagonality has nearly the same curve; anyway, the percentage of hexagonality has the decreased rate as 1.08%/year which is resemblance to the rate of ECD reduction. Those data affirm that the different endothelial qualifications such as ECD and hexagonality reach the evaluation dimensions consistency which coincide with Joo et al.'s publications [23].

### 4.4. The Cell Size and Morphology Changes upon Stages of Human Development

The size and hexagon of endothelial cell area can be the unique property for the human development [24]. Meanwhile, Radman et al.'s literatures have reported that the sharp of the human endothelial be associated with the age growth stage, even that so-called childhood obesity criterion can be realized fiercely different with adolescence and earlier adulthood [25]. From our obtained data of the age correlative cell size criterions, the 4-8 and 9-12 age groups are the same level (childhood); the 13-16 and the 17-20 age group (puberty) have the same tender; meanwhile, the 21-25 and the 26-30 age group (adulthood) presses the consistency curve. From the data of human corneal endothelial variations within the dimension that manifests that the 4-12 year phrase should name as the childhood, the 13-20 year group can be treat as the puberty, and the age more than 21 year could be attributed as the adulthood.

### 4.5. The Standard Approved Proposal for Endothelial Estimation

Endothelial cell density (ECD) is an important parameter for assessing the endothelial function and health. Polymegathism, marked by the coefficient of variation in cell surface (CV), and pleomorphism, determinate by the ratio of Hexagonal cells (HEX), are other vital parameters that dispose stress to the endothelial [26]. The Food and Drug Administration of USA recommends the key method of specular microscopy as the “gold standard;” meanwhile, it is used by essentially each technical reading center [27, 28]. The multiply-center method allows the inclusion of the superficial cells and increases the NUM (the number of analyzed cells) and should be recommended for cases with least unambiguously cell numbers [29], and our eye hospital groups make up of more than thousands bunch of hospitals that assure the random assigned center method reliable and homogenization which requires the handle-automatic boundary tracing of the contiguous cell fulfills the cell-center dotting and should be more time consuming than the traditional center method. Actuality, the least contiguous countable cells in an image of our study are 86 which are more trustworthy than previous literatures [30, 31].

### 4.6. The Valuable of HEX and *S*_min_ Index

Our observe trial has enrolled more than 10 thousand age-appropriated volunteers (14,670, 6 age groups) and massive information collection ensuring the accurate and reliable of those inferred conclusions. The quantitative indicators such as the CCT, ECD, and HEX had the significant negative association with age by multivariate regressive analysis (*p* = 0.001 or *p* < 0.001) which confirmed with Jha's literature [32]. Meanwhile, the corneal endothelial variability quotas such as the CV, *S*_min_, *S*_max_, *S*_avg_, and *S*_sd_ express the opposite correlation (*p* < 0.001). Anyway, the age relative CCT data can be the least statistic valuable influence compared with all other aspects. The CV of HE is -0.59 which is a bit more exaggerated index for the intention curve, so that from the regression equation index dimension, the HEX can be the most sensitive index for the morphology of single endothelial the same as Abdellah's commends [10]. The CV of the size of minimal cell is 1.17 which is the least among all these polymegathism assessment quotas; from the cell size index dimension, the minimal cell size should be the most specific index for the variation of endothelial.

### 4.7. Limitations

Our study focuses on the normal Chinese Han endothelial biology development of the childhood to early adults. Although the enrollment volunteer number is large and the gender bias is under control, the distinctive ECD seems higher than those all-periods' human beings' ECD decrease rate. Meanwhile, this characteristic should be test and verified on other ethnicities or/and age groups' population for further considerations.

## 5. Conclusion

In conclusion, this study provides the first characterization of specular microscopy-based CCT and CEC in 4 to 30 years healthy Chinese Hans' corneal endothelial subjects.

The CCT was decreased during this period but without statistic significant. The ECD decrease rate is 1.02%/year from the childhood to the earlier adulthood of Chinese Han nationality. The corneal endothelial variations within the scope that manifests that the aged 4-12, 13-20, and more than 21 year old can be redefine as the childhood, puberty, and adulthood.

Although the Topcon Noncon Specular microscopy can provide the eight evaluation indexes, quality, and quantity, the percentage of regular hexagonal cells (HEX) can be the most sensitive one for the endothelial polymorphisms. The cell size of minimal cell (*S*_min_) should be the most specificity assessment indicators for the variability.

## Figures and Tables

**Figure 4:**
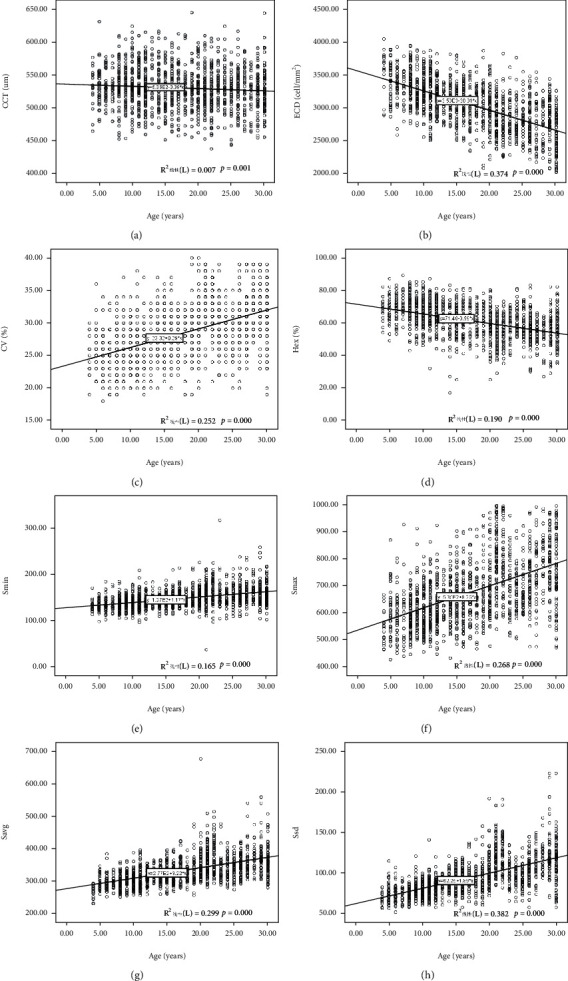
Eight parameters in different study groups. (a) Central corneal thickness. (b) Endothelial cell density. (c) Coefficient of variability. (d) Hexagonality. (e) Size of minimal cell. (g) Average cell size. (h) Size standard deviation.

**Table 1:** Parameters of corneal thickness and endothelial characteristics in normal Chinese Han children and youngsters.

Parameters	Minimal value	Maximal value	Average (mean ± SD)
Counted cells (cells)	86	394	221.71 ± 63.21
CCT (*μ*m)	437	644	529.94 ± 31.53
ECD (cells/mm^2^)	2031	4074	3051.28 ± 375.49
CV (%)	18	40	28.34 ± 4.36
HEX (%)	17	89	61.21 ± 10.29
*S*_min_ (S/*μ*m^2^)	37	316	147.79 ± 21.94
*S*_max_ (S/*μ*m^2^)	428	997	678.29 ± 120.96
*S*_avg_ (S/*μ*m^2^)	231	678	332.74 ± 44.62
*S*_sd_ (S/*μ*m^2^)	52	223	95.02 ± 23.17

Note: CCT: central corneal thickness; ECD: endothelial cell density; CV: coefficient of variability; HEX: hexagonality; *S*_min_: size of minimal cell; *S*_max_: size of maximal cell; *S*_avg_: average cell size; *S*_sd_: size standard deviation.

**Table 2:** Parameters of corneal thickness and endothelial characteristics in different age groups of normal Chinese Han children and youngsters.

Parameters	Ages 4-8	Ages 9-12	Ages 13-16	Ages 17-20	Ages 21-25	Ages 26-30	Hc/F	*p*
Counted cells (*n*)	258.45 ± 61.97	246.09 ± 60.84	218.73 ± 56.53	211.27 ± 58.63	200.21 ± 59.41	199.15 ± 56.86	43.262	<0.001
CCT (*μ*m)	532.76 ± 28.46	532.96 ± 31.00	531.44 ± 33.08	529.48 ± 32.36	527.63 ± 32.40	526.16 ± 31.35	2.117	0.061
ECD (cells/mm^2^)	3,407.40 ± 279.84	3,246.36 ± 271.53	3,129.45 ± 269.88	3,031.46 ± 315.56	2,838.22 ± 286.72	2,750.39 ± 346.17	175.896	<0.001
CV (%)	25.28 ± 3.12	25.87 ± 3.10	27.70 ± 3.53	28.34 ± 3.75	30.88 ± 4.20	31.14 ± 4.19	115.119	<0.001
HEX (%)	258.45 ± 61.97	246.09 ± 60.84	218.73 ± 56.53	211.27 ± 58.63	200.21 ± 59.41	199.15 ± 56.86	79.334	<0.001
*S*_min_ (S/*μ*m^2^)	134.95 ± 14.05	139.14 ± 15.30	143.50 ± 15.12	149.58 ± 18.05	156.62 ± 26.65	159.55 ± 24.36	61.226	<0.001
*S*_max_ (S/*μ*m^2^)	590.41 ± 80.17	616.72 ± 83.27	646.13 ± 92.69	680.44 ± 106.65	746.06 ± 119.64	763.14 ± 116.97	121.240	<0.001
*S*_avg_ (S/*μ*m^2^)	295.00 ± 23.99	310.24 ± 26.41	321.90 ± 28.31	340.34 ± 44.35	355.73 ± 44.72	364.28 ± 45.37	133.426	<0.001
*S*_sd_ (S/*μ*m^2^)	74.83 ± 9.93	80.24 ± 10.94	89.19 ± 13.27	97.04 ± 19.04	109.60 ± 21.99	114.01 ± 24.56	200.588	<0.001

Note: CCT: central corneal thickness; ECD: endothelial cell density; CV: coefficient of variability; HEX: hexagonality; *S*_min_: size of minimal cell; *S*_max_: size of maximal cell; *S*_avg_: average cell size; *S*_sd_: size standard deviation.

**Table 3:** Comparison of variables pertaining to the corneal thickness and endothelial characteristics between male and female in normal Chinese children and youngsters.

Parameters	Male (mean ± SD)	Female (mean ± SD)	*t*-test	*p* value
CCT (*μ*m)	529.52 ± 31.89	530.34 ± 31.19	0.248	0.618
ECD (cells/mm^2^)	3060.42 ± 383.64	3042.33 ± 367.36	0.851	0.356
CV (%)	28.14 ± 4.12	28.53 ± 4.58	2.891	0.089
HEX (%)	61.48 ± 10.11	60.95 ± 10.47	0.990	0.320
*S*_min_ (S/*μ*m^2^)	147.39 ± 23.63	148.19 ± 20.16	0.481	0.488
*S*_max_ (S/*μ*m^2^)	681.53 ± 128.06	675.11 ± 113.57	1.032	0.310
*S*_avg_ (S/*μ*m^2^)	330.64 ± 45.27	334.80 ± 43.90	3.188	0.074
*S*_sd_ (S/*μ*m^2^)	95.11 ± 24.43	94.94 ± 21.87	0.019	0.891

Note: SD: standard deviation; CCT: central corneal thickness; ECD: endothelial cell density; CV: coefficient of variation in cell area; HEX: hexagonal cells; *S*_min_: size of minimal cell; *S*_max_: size of maximal cell; *S*_avg_: average cell size; *S*_sd_: size standard deviation.

**Table 4:** Correlation between age and variables pertaining to the corneal thickness and endothelial characteristics in normal Chinese Han children and youngsters.

Parameters	Pearson's correlation coefficient (*r*)	*p* value
CCT (*μ*m)	−0.084	0.001
ECD (cells/mm^2^)	−0.612	<0.001
CV (%)	0.502	<0.001
HEX (%)	−0.436	<0.001
*S*_min_ (S/*μ*m^2^)	0.406	<0.001
*S*_max_ (S/*μ*m^2^)	0.518	<0.001
*S*_avg_ (S/*μ*m^2^)	0.547	<0.001
*S*_sd_ (S/*μ*m^2^)	0.618	<0.001

Note: SD: standard deviation; CCT: central corneal thickness; ECD: endothelial cell density; CV: coefficient of variation in cell area; HEX: hexagonal cells; *S*_min_: size of minimal cell; *S*_max_: size of maximal cell; *S*_avg_: average cell size; *S*_sd_: size standard deviation.

## Data Availability

The datasets used and/or analyzed during the present study are available from the corresponding author on reasonable request.
